# Implementation of a practical sand constitutive model coupled with the high cycle accumulation framework in PLAXIS

**DOI:** 10.1016/j.mex.2025.103183

**Published:** 2025-01-25

**Authors:** Pishun Tantivangphaisal, David M.G. Taborda, Stavroula Kontoe

**Affiliations:** a Department of Civil and Environmental Engineering, Imperial College London, Exhibition Road, London SW7 2AZ, United Kingdom; b Department of Civil Engineering, University of Patras, Greece

**Keywords:** Cyclic loading, Constitutive model, Sand behaviour, High cycle accumulation, Long-term loading, Implementation, Practical state parameter enhanced hypoelastic Mohr-coulomb sand constitutive model extended with the High Cycle Accumulation framework

## Abstract

A modification of the high-cycle accumulation (HCA) framework coupled with a practical constitutive model for sands and its numerical implementation as a user-defined soil model in PLAXIS is presented. The implemented model is compared against data from the original high-cyclic tests in Karlsruhe fine sand and more recent laboratory tests in Dunkirk sand. A reference 15 MW offshore wind turbine monopile foundation subject to lateral cyclic wave loading is used in an engineering design scenario at three different load levels to verify the current numerical implementation.

Details include:

• Modifications made to the HCA framework to couple it with a practical sand constitutive model,
• Implementation of an efficient workflow to switch between low and high cycle constitutive equations in PLAXIS, and
• Verification of the implementation at single element and boundary value problem scales.

## Graphical abstract

**Figure:**
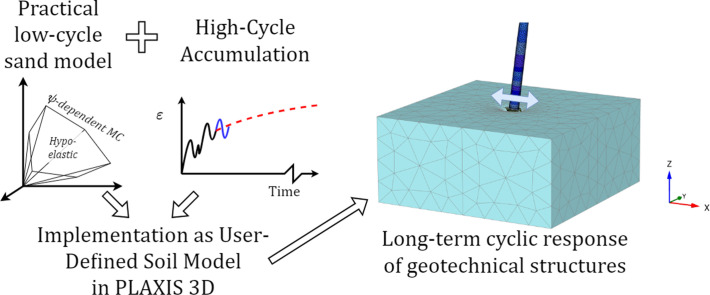

Specifications table

**Table:** 

Subject area:	Engineering
More specific subject area:	Computational geomechanics
Name of your method:	Practical state parameter enhanced hypoelastic Mohr-coulomb sand constitutive model extended with the High Cycle Accumulation framework
Name and reference of original method:	Niemunis, A., Wichtmann, T. & Triantafyllidis, Th. (2005) A high-cycle accumulation model for sand. Computers and Geotechnics. 32 (4), 245–263. doi:10.1016/j.compgeo.2005.03.002. Wichtmann, T., Niemunis, A. & Triantafyllidis, T. (2013) On the “elastic stiffness” in a high-cycle accumulation model — continued investigations. Canadian Geotechnical Journal. 50 (12), 1260–1272. doi:10.1139/cgj-2013–0037. Staubach, P., Machacek, J., Tschirschky, L. & Wichtmann, T. (2022) Enhancement of a high‐cycle accumulation model by an adaptive strain. International Journal for Numerical and Analytical Methods in Geomechanics. 46, 315–338. doi:10.1002/nag.3301. Taborda, D.M.G., Pedro, A.M.G. & Pirrone, A.I. (2022) A state parameter-dependent constitutive model for sands based on the Mohr-Coulomb failure criterion. Computers and Geotechnics. 148, 104,811. doi:10.1016/j.compgeo.2022.104811.
Resource availability:	Bentley Systems (2024) PLAXIS 3D. https://www.seequent.com/products-solutions/plaxis-3d/.

## Background

The consideration of long-term, regular cyclic actions, such as environmental (wind, wave, temperature, etc.) or anthropogenic loading (road traffic, rail, etc.) is often necessary to ensure the stability and serviceability of engineered structures throughout its full design life. When significant interaction occurs between a structure and the ground on, or in, which it is founded, the mechanical behaviour of the surrounding soil subject to such cyclic loads often governs the whole-life engineering design.

In offshore geotechnical engineering, design guidelines [1,2] now routinely recommend the use of 3D finite element (FE) analyses in the design of offshore wind turbine foundations. The problem geometry of the dominant foundation type, namely large diameter, low length-to-diameter (L/D) monopiles, means that long-term regular cyclic wind and wave loading is resisted largely via lateral transmission of stresses to the surrounding soil mass, rather than the pile-soil interface as in axially loaded piles. Considering a typical 30-year design life with an average frequency of 0.1 Hz would require the design of such structures to account for $10^{8}$ loading cycles.

The additional challenge of high computational costs associated with the simulation of these loading conditions adds to that of constitutive models capable of capturing the highly non-linear and history-dependent mechanical stress-strain behaviour of soils. Additionally, geo-materials are natural in origin and always require site-specific calibration. Ease-of-calibration is thus hugely relevant for any model to be of practical use in design problems.

This work presents modifications to the high cycle accumulation (HCA) framework [3] to model the mechanical behaviour of sandy soils under long-term cyclic loading and its implementation in the PLAXIS software package commonly used in geotechnical engineering design practice [4]. The original framework consists of two major constitutive ingredients, a low-cycle and a high-cycle part. The original low-cycle model is replaced with a practice oriented constitutive model for sands [5]. Empirical functions used in the high-cycle part are revised to better capture trends observed in laboratory experiments on Dunkirk sand [6], and then further modified in the present numerical implementation to ensure stability and compatibility with the selected low-cycle model.

A highly pertinent engineering application in tackling the global climate crisis, namely the design of offshore wind turbine foundations resisting long-term wind and wave loading, is used to illustrate this method of analysis and its implementation in the PLAXIS software package. Although an example from offshore engineering is selected, the method can be used for any long-term cyclic sand-structure interaction problem.

## Method details

Niemunis et al. [3] proposed that the mechanical behaviour of soils subject to cyclic loading can be characterized by a series of empirical functions relating the change in average strain state with number of cycles, cyclic strain amplitude, average stress state and cyclic loading history, supported by an initial set of laboratory tests from Wichtmann [7]. The fundamental assumption of this method is that the characterisation of these variables at the level of an integration point in a finite element enables the accurate modelling of their effects on the structure considered in a boundary value problem. To compute these boundary value problem, two distinctive constitutive schemes, originally termed “calculation strategies”, are used. The original terminology of “implicit” and “explicit” calculation phases can cause confusion as the terms also describe numerical integration methods which are independent of the constitutive schemes. Instead, “low-cycle” and “high-cycle” analysis phases are used in this work.

For low-cycle phases of an analysis, a conventional elasto-plastic constitutive model is adopted. At any material point in a continuum representing a body of soil (integration point in a finite element), given some total strain increment $\left\{\Delta \varepsilon^{tot}\right\}$, the constitutive model is required to return the plastic strain increment $\left\{\Delta \varepsilon^{pl}\right\}$ and the tangent elastic stiffness matrix $\left[D^{ela}\right]$ to predict a corresponding conventional stress increment $\left\{\Delta \sigma^{\mathrm{con}}\right\}$.

(1) $$\begin{array}{c} \left\{\Delta \sigma^{\mathrm{con}}\right\}=\left[D^{\mathrm{ela}}\right]\left(\left\{\Delta \varepsilon^{\mathrm{tot}}\right\}-\left\{\Delta \varepsilon^{\mathrm{pl}}\right\}\right) \end{array}$$

In this framework, during high-cycle analysis phases, total strain increments $\left\{\Delta \varepsilon^{tot}\right\}=\Delta N\cdot \left\{\frac{\partial \varepsilon^{tot}}{\partial N}\right\}$ no longer denote conventional physical increments of strain, but rather the change in the average strain state during a package of ΔN cyclic loads. Accumulated strain increments are given by the rate of change in the average strain state $\left\{\frac{\partial \varepsilon^{acc}}{\partial N}\right\}$ given some constant average stress boundary conditions. The equivalent HCA tangent elastic stiffness matrix $\left[D^{HCA,ela}\right]$ governs the relationship between strain accumulation and stress relaxation. These two terms form the core of the HCA framework. Finally, plastic strain increments $\left\{\Delta \varepsilon^{pl}\right\}=\Delta N\cdot \left\{\frac{\partial \varepsilon^{pl}}{\partial N}\right\}$ ensure the stress state persists on the underlying model's yield surface in stress space. The accumulated stress change during high-cycle phases predicted by the constitutive model is $\left\{\Delta \sigma^{\mathrm{acc}}\right\}$, which is evaluated as:

(2) $$\begin{array}{c} \left\{\Delta \sigma^{\mathrm{acc}}\right\}=\Delta N\cdot \left\{\frac{\partial \sigma^{\mathrm{acc}}}{\partial N}\right\}=\Delta N\cdot \left[D^{\mathrm{HCA},\mathrm{ela}}\right]\left(\left\{\frac{\partial \varepsilon^{\mathrm{tot}}}{\partial N}\right\}-\left\{\frac{\partial \varepsilon^{\mathrm{pl}}}{\partial N}\right\}-\left\{\frac{\partial \varepsilon^{\mathrm{acc}}}{\partial N}\right\}\right) \end{array}$$

**Fig. 1:**
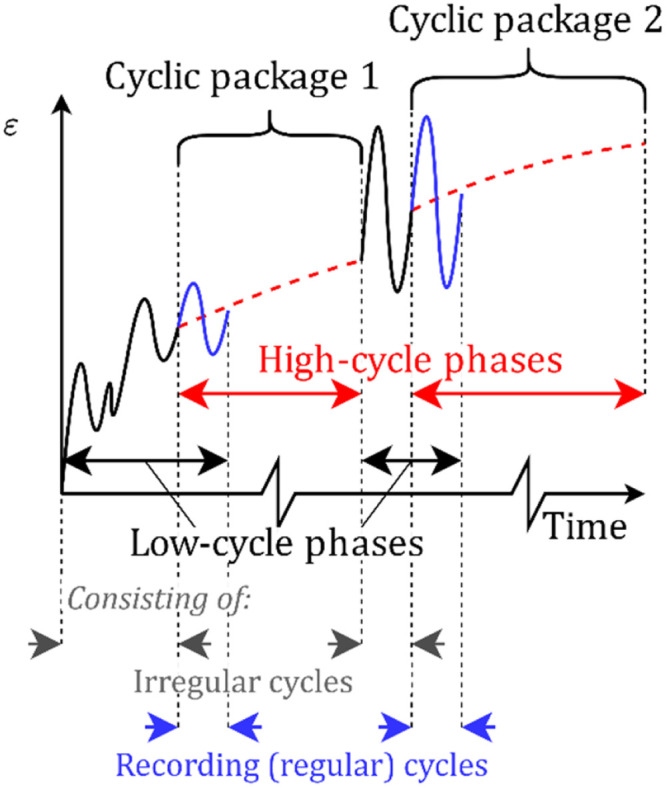
Schematic of the HCA framework at a material point illustrated for an arbitrary strain measure.

The types of analysis phases are illustrated in Fig. 1 for an arbitrary scalar strain measure. Low-cycle analysis phases are also separated into irregular phases (where the state variable $\varepsilon^{ampl}$ is not updated and shown in black in Fig. 1) and regular recording phases (where $\varepsilon^{ampl}$ is updated and shown in blue in Fig. 1).

In their large database of laboratory experiments on clean sands, Wichtmann [7,8] found that the magnitude of the strain accumulation rate $∥\{\frac{\partial \varepsilon^{acc}}{\partial N}\}∥$ at any material point is generally dependent on:

1. A scalar measure of cyclic strain amplitude $\varepsilon^{ampl}$,
2. The average void ratio $e_{av}$,
3. The average mean effective stress $p_{av}^{\prime }$,
4. A measure of the average stress ratio $\frac{J_{av}}{p_{av}^{\prime }}$, and
5. A measure of the cyclic history $g_{cyc}$.

where $p^{\prime },\;J,\;\theta$ are the conventional soil mechanics invariants of the Cauchy stress tensor $\sigma_{ij}$, and the subscript ${}_{av}$ denotes the average value during regular cyclic loading.

The magnitude of the rate of strain accumulation is composed of the product of empirical functions taking these factors into account:

(3) $$\left|\left|\left\{\frac{\partial \varepsilon^{acc}}{\partial N}\right\}\right|\right|=\frac{\partial g_{cyc}}{\partial N}\cdot f_{e}f_{p}f_{Y}$$

The form of $f_{p}$ from Niemunis et al. [3] remains unchanged in the current implementation:

(4) $$f_{p}=\exp\left(-C_{p}\cdot \left(\frac{p_{av}^{\prime }}{101.3\left[kPa\right]}-1\right)\right)$$

The cyclic history function $g_{cyc}$ and its rate of change are modified to a power-law function as it best fit observations of Dunkirk sand laboratory tests [6]. $g_{cyc}$ is defined as the product of the amplitude and cycle-number factors:

(5) $$g_{cyc}=f_{N}\cdot f_{ampl}$$

where:

(6) $$f_{ampl}={\left(\frac{\min\left(\varepsilon^{ampl},\;\varepsilon^{lim}\right)}{10^{-4}}\right)}^{C_{ampl}}$$

and the scalar cyclic strain amplitude $\varepsilon^{ampl}$ is defined in Niemunis et al. [3].

$f_{N}$ is the newly proposed power-law:

(7) $$f_{N}=C_{N1}{\left(1+N\right)}^{C_{N2}}-C_{N1}$$

To derive the evolution of the cyclic history variable, $\frac{\partial g_{cyc}}{\partial N}$, $f_{ampl}$ is assumed constant during an arbitrary cyclic load package from $N_{0}$ to N, i.e. $\frac{\partial f_{ampl}}{\partial N}=0$ and therefore:

(8) $$\frac{\partial g_{cyc}}{\partial N}=\frac{\partial f_{N}}{\partial N}\cdot f_{ampl}=\left(C_{N1}C_{N2}{\left(1+N\right)}^{\left(C_{N2}-1\right)}\right)\cdot f_{ampl}$$

An arbitrary increment in $g_{cyc}$ is the integral of this quantity over the considered load package (i.e. from $N_{0}$ to N):

(9) $$\Delta g_{cyc}=g_{cyc}\left(N\right)-g_{cyc}\left(N_{0}\right)=\int_{N_{0}\;}^{N}f_{ampl}\cdot \left(C_{N1}C_{N2}{\left(1+N\right)}^{\left(C_{N2}-1\right)}\right)dN$$

where $g_{cyc}\left(N_{0}\right)$ is the value of $g_{cyc}$ at the last update of the cyclic strain amplitude $\varepsilon^{ampl}$ (and hence $f_{ampl}$). Integrating and rearranging Eq. (9) with respect to N results in:

(10) $$N={\left[\frac{g_{cyc}\left(N\right)-g_{cyc}\left(N_{0}\right)}{f_{ampl}\cdot C_{N1}}+{\left(1+N_{0}\right)}^{C_{N2}}\right]}^{\frac{1}{C_{N2}}}-1$$

Finally, on substitution into Eq. (8):

(11) $$\frac{\partial g_{cyc}}{\partial N}=f_{ampl}\cdot \frac{\partial f_{N}}{\partial N}=f_{ampl}\cdot C_{N1}C_{N2}{\left(\frac{g_{cyc}\left(N\right)-g_{cyc}\left(N_{0}\right)}{f_{ampl}\cdot C_{N1}}+{\left(1+N_{0}\right)}^{C_{N2}}\right)}^{\left(1-\frac{1}{C_{N2}}\right)}$$

The void ratio dependency function $f_{e}$ is also modified to be bounded between 0 and 1 and to prevent numerical issues at excessively low or high void ratios:

(12) $$f_{e}=\left\{ \begin{array}{c} 1,\;e\geq e_{max}\\ 0,\;e<e_{max}-1/C_{e}\\ 1-C_{e}\left(e_{max}-e\right),\;\text{otherwise} \end{array}\right.$$

The exponential relationship is retained for the stress ratio function $f_{Y}$ but normalisation is made consistent with the failure criterion of the selected low-cycle model:

(13) $$f_{Y}=\exp\left(C_{Y}\cdot \frac{\frac{J_{av}}{p_{av}^{\prime }}}{g\left(\phi_{mag}^{\prime },\theta_{av}\right)}\right)$$

where:

$g(\phi_{mag}^{\prime },\;\theta_{av})$ is the available strength defined by the Mohr-coulomb failure criterion expressed as a stress ratio, and

$C_{ampl},C_{N1},C_{N2},C_{e},e_{max},C_{p},C_{Y},\phi_{mag}^{\prime },\varepsilon_{lim}$ are material parameters calibrated against laboratory tests.

High-cycle specific state variables are the average stresses $p_{av}^{\prime },\;J_{av},\;\theta_{av}$, average void ratio $e_{av}$, cyclic history during the current high-cycle phase $g_{cyc}$, previous cyclic history $g_{cyc}\left(N_{0}\right),\;N_{0}$, and strain amplitude $\varepsilon^{ampl}$.

**Fig. 2:**
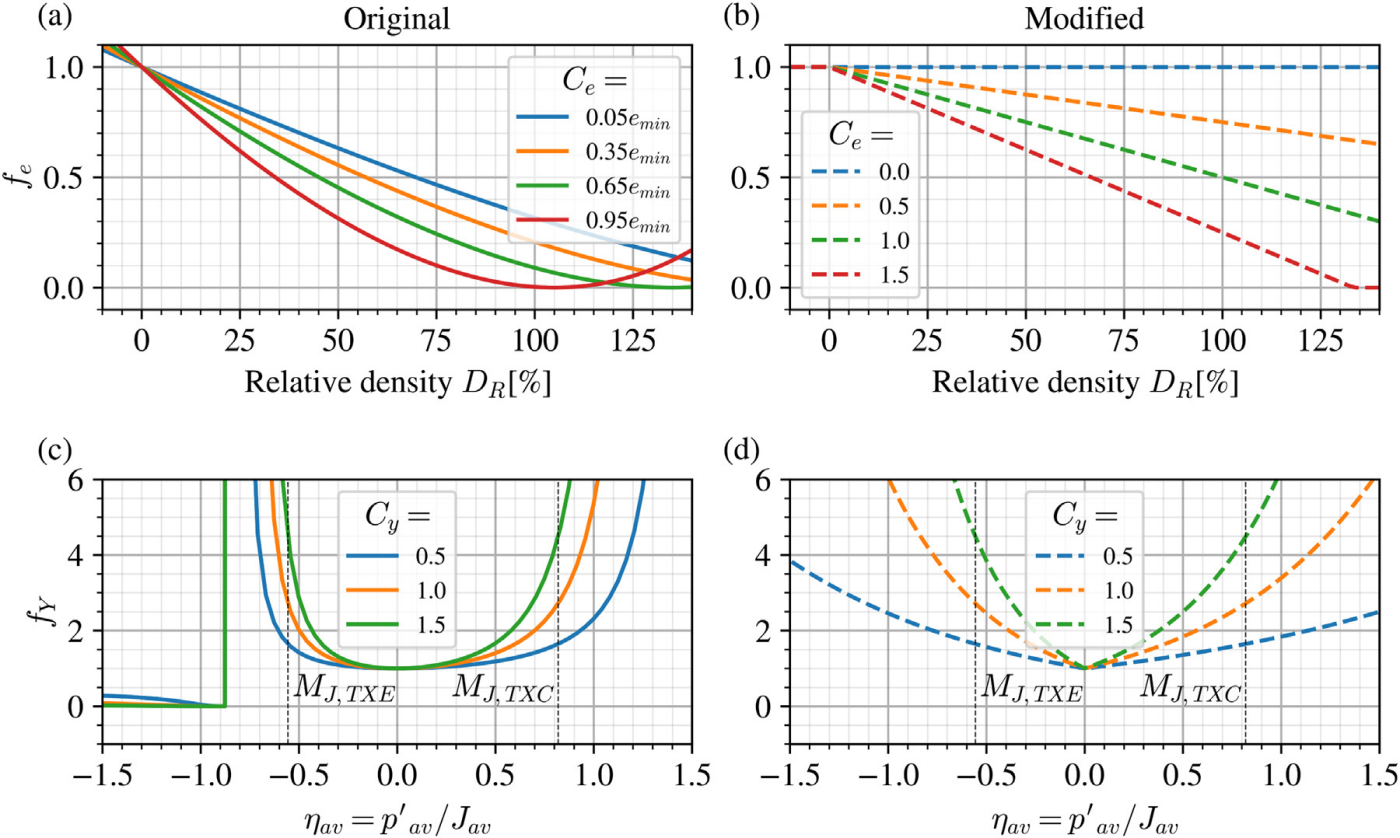
Illustration of the (a) original and (b) modified forms of ***f_e_*** and (c) original and (d) modified forms of ***f_Y_*** for improved numerical stability.

Fig. 2 illustrates the modified forms of $f_{e}$ and $f_{Y}$ compared to those originally proposed in Niemunis et al. [3] and adopted in Wichtmann et al. [9] and Machacek et al. [10]. As global integration error controls available to the user vary from software to software, the current implemented forms ensure that:

1. In case of densification beyond some arbitrary minimum void ratio or maximum relative density, the predicted void ratio dependency does not reverse as seen in Fig. 2(a) for $C_{e}=0.95\;e_{min}$.
2. If the current stress ratio at any integration point exceeds the available strength defined by $\phi_{mag}^{\prime }$, large jumps (discontinuities) caused by the original Matsuoka-Nakai normalisation can be avoided. This is seen on the left-hand side of Fig. 2(c).

A final modification is made to decouple strain accumulation direction with magnitude. This unit accumulation direction vector $\left\{\vec{m}\right\}=\left\{\frac{\partial \varepsilon^{acc}}{\partial N}\right\}/\left|\left|\{\frac{\partial \varepsilon^{acc}}{\partial N}\}\right|\right|$ is normal to the standard Modified Cam Clay surface fitted through the current average stress state with its size defined by material parameter $\phi_{dir}^{\prime }$. This modifies the original proposal to use the angle of shearing resistance at critical state in Wichtmann et al. [11], and allows direct calibration of strain accumulation direction using laboratory observations without a need to quantify anisotropy [9].

### Updating of high-cycle model state variables

Stresses $p_{av}^{\prime },J_{av},\theta_{av}$ are updated during a high-cycle phase as part of the global finite element solution scheme. The cyclic history variable $g_{cyc}$ evolves as derived in Eq. (11). $g_{cyc}\left(N_{0}\right)$ and $N_{0}$ are initialised as zero at the start, then re-initialised at the start of any given high-cycle analysis phase, remaining constant until re-initialised in the next high-cycle phase. $\varepsilon^{ampl}$ is also constant during each high-cycle analysis phase and initialised for each integration point using the strain path recorded in the previous low-cycle recording phases. Changes in void ratio are addressed later to account for total, plastic and accumulated volumetric strain increments depending on the type of integration step.

### Updating of low-cycle model state variables during high-cycle analysis phases

Approximations made by the HCA framework to overcome the computational cost of large numbers of loading cycles include the assumption of a negligible change to the single-cycle strain amplitude $\varepsilon^{ampl}$ over several cycles. Small changes to $\varepsilon^{ampl}$ occurring over large numbers of cycles can be taken into account 1) by re-engaging the low-cycle model or 2) via an adaptive update approach [12].

Simulations of multiple load packages of varying sizes must utilise the former approach and re-engage the low-cycle model over a single recording cycle (average-peak-trough-average) in order to update the high-cycle state variable $\varepsilon^{ampl}$. This approach, however, requires tracking the low-cycle model's state variables without explicitly using their original evolution rules. Although the second adaptive strain update approach investigated by Staubach et al. [12] cannot account for load packages that vary in size sequentially, its underlying philosophy has merit and is examined to help derive an appropriate evolution rule for the state variables in the current low-cycle model during accumulation phases.

The adaptive strain approach assumes that strain amplitude is inversely proportional to the soil stiffness, following a simple logic that under the same stress cycle a stiffer material experiences smaller strains. Thus, instead of the re-engaging the low-cycle model to update the strain amplitude state variable, this update is simply the ratio of previous-to-current stiffness tensor norms. Staubach et al. [12] suggests that both a ‘tangent’ and ‘secant’ measure of stiffness can be used, opting for the ‘secant’ measure as their definition of ‘tangent’ is step size dependent. It is presumed here that what is meant by the ‘secant’ measure is in fact the elastic stiffness tensor of the underlying low-cycle model at the start of an equivalent update cycle. Denoting the incremental rank-4 tangent elastic tensor as $C_{ijkl}$, i.e. $\Delta \sigma_{ij}=C_{ijkl}\Delta \varepsilon_{kl}^{ela}$, an update of $\varepsilon^{ampl}$ between two cyclic packages with subscripts 1 and 2 is:

(14) $$\varepsilon_{2}^{ampl}=\frac{{∥C_{ijkl}∥}_{1}}{{∥C_{ijkl}∥}_{2}}\varepsilon_{1}^{ampl}=\frac{{\left(\sqrt{C_{ijkl}C_{ijkl}}\right)}_{1}}{{\left(\sqrt{C_{ijkl}C_{ijkl}}\right)}_{2}}\varepsilon_{1}^{ampl}$$

Using an isotropic elastic stiffness in the current low-cycle model [5], Eq. (14) can be re-written using the tangent elastic shear and bulk moduli. Assuming a constant small-strain Poisson's ratio, this can be further written as a ratio between the tangent elastic shear moduli at the start of cyclic package 1 and 2 as:

(15) $$\varepsilon_{2}^{ampl}=\frac{{\left(\sqrt{20G_{tan}^{2}+9K_{tan}^{2}}\right)}_{1}}{{\left(\sqrt{20G_{tan}^{2}+9K_{tan}^{2}}\right)}_{2}}\varepsilon_{1}^{ampl}=\frac{G_{tan_{1}}\left(e_{1},p_{1}^{\prime },E_{d_{1}}^{*}\right)}{G_{tan_{2}}\left(e_{2},p_{2}^{\prime },E_{d_{2}}^{*}\right)}\varepsilon_{1}^{ampl}$$

When re-engaging the underlying low-cycle model, the update to $∥C_{ijkl}∥$ is thus assumed to only be a function of changes in *e* and *p'*. Therefore, any history variables required by the underlying model's hypoelastic formulation to track reversals [13,14], such as measures derived from history of the deviatoric strain invariant *E_d_*, namely the tensorial lag and reversal strain states, are modified to evolve together with the total input strain step during high-cycle analysis phases.

### Numerical implementation in PLAXIS

The low-cycle model coupled with the HCA framework is implemented as a User-Defined Soil Model (UDSM) in PLAXIS [4]. As switching between different constitutive equations is required, the in-built construction phasing and multi-branch calculation functionalities are utilised. Five distinct PLAXIS construction phase types (Table 1) are implemented using an input flag termed ‘special option’ in the software graphical user interface (GUI) to control which components of the framework should be employed at different parts of the full analysis.

**Table 1:** Construction phase types and corresponding flags implemented in PLAXIS.

Flag	Task	Load boundary conditions	Strain state files I/O		Constitutive relation	
			Write	Read	Elastic / Accumulation	Plastic
1	Any loading	Any	N	N	Low-cycle model	
2	Recording cycle	Ave-Peak-Trough-Ave	Y	N	Low-cycle model	
3	End of recording	No change	Y	N	Low-cycle model	
4	HCA state variable initialisation	No change	N	Y	Low-cycle model	
5	HCA accumulation	No change	N	N	HCA with low-cycle state variable evolution	Low-cycle model

### Low-cycle, recording phases implementation

**Fig. 3:**
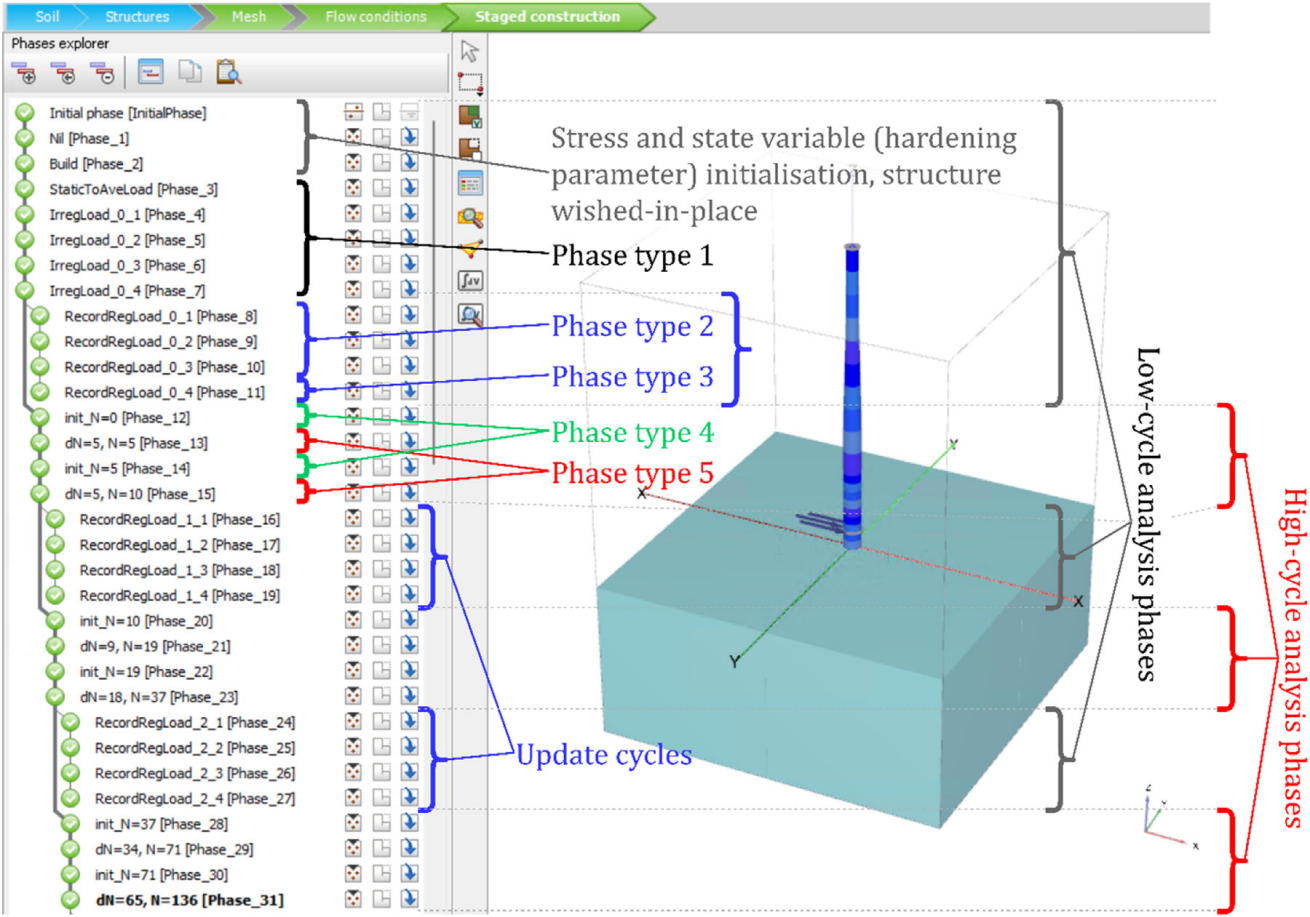
Annotated screenshot of the construction phase set-up for the current implementation of an HCA analysis.

The same strain step filter described in Niemunis et al. [3] is used in construction phase type 2. For both static (modified Newton-Raphson) and dynamic (Newmark) global integration schemes, the strain state at the start of each global step is passed to the filter and assessed. Multiple type 2 construction phases or a single dynamic construction phase can be used to simulate a single recording load cycle. The end of recording is signified by assigning an additional ‘null phase’ (type 3) immediately after the last type 2 phase, with inputs of zero total time elapsed and no changes to boundary conditions. For each analysis instance, recorded strains are stored in long-term storage in a temporary directory with one file per integration point. Contrary to previous HCA studies [9], the definition of N=0 for accumulation coincides with the start of the first regular cycle at the current load level and is concurrent to any regular or update cycle. This removes the issue of inadvertently double counting strain predicted by the low-cycle model over an update cycle on top of the HCA prediction. The construction phase branching in PLAXIS is utilised where update cycles (phase type 2 and 3) and accumulation phase types 4 and 5 are computed sequentially but are parallel in the analysis timeline. A typical screenshot of the software GUI with an analysis set-up for multiple low-cycle and high-cycle analysis phases is depicted in Fig. 3. The annotations illustrate a pseudo-static analysis where the rate of loading is slow and inertial forces considered negligible. As shown, multiple static construction phases for irregular and regular recording cycles can be used in the current implementation.

### HCA initialization phase

Phase type 4 must be used immediately before each HCA accumulation (type 5) to initialise HCA state variables by reading recorded strains or updating $\varepsilon^{ampl}$ following the adaptive strain approach. For the former, a scalar strain amplitude is calculated from the six-dimensional strain path for each integration point [3,15] if the strain file contains two or more strain steps. Each strain file is deleted on reading to make way for future update cycles. Finally, an integer state variable is used as an HCA phase counter. This is incremented by one at the start of each phase type 4 for use in the subsequent accumulation phase. Multiple pairs of type 4 and type 5 phases (respectively in green and red text in Fig. 3) can be used for a single high-cycle load package. This implementation detail overcomes the issue of having limited step size and global error controls, as well as a hard cap of 10,000 global integration steps per construction phase in the PLAXIS software.

### Accumulation or high-cycle phases

A text-based input file is used to specify $N_{0,i},\;N_{total,i}$ and $T_{total}$ for each accumulation phase. These respectively denote, for any phase i, the starting value of $N_{0}$, total number of cycles and total time elapsed (i.e. the specified ‘time interval’ in the graphical user interface input for construction phase settings). This ensures that each time step $\Delta T_{kernel}$ passed from the global solver to the user-defined soil model constitutive integrator can be converted to the correct cycle number increment ΔN for the constitutive relation in Eq. (2) by:

(16) $$\Delta N=N_{total,i}\cdot \frac{\Delta T_{kernel}}{T_{total,i}}$$

A second text file is used for material specific HCA parameters. Each PLAXIS soil material with a specified set of HCA parameters is assigned a corresponding integer flag as a ‘mechanical property’ in the PLAXIS graphic user interface (GUI).

A two-stage explicit modified-Euler scheme with automatic sub-stepping [16] and root finding algorithm [25] is used to integrate over the non-linear elastic stiffness matrix, strain accumulation, and plastic strain predictors to return stress changes at each integration point. If the current stress state is not on the yield surface, an elastic step is first assumed and sub-stepped until a normalised convergence criterion is met for both forward and backward accumulated strain and stress changes. Yield surface intersection is determined from this stress change. If elastoplastic, a modified total strain step $\left\{\Delta \varepsilon^{tot}-\Delta \varepsilon^{acc}\right\}$ is passed to the conventional elastoplastic integration scheme. The void ratio update, which normally depends on the total strain step, is now modified to be updated using $\left\{\Delta \varepsilon^{acc}\right\}$ during the elastic portion of the step and $\left\{\Delta \varepsilon^{tot}\right\}-\left\{\Delta \varepsilon^{acc}\right\}$ when the material is elasto-plastic.

**Fig. 4:**
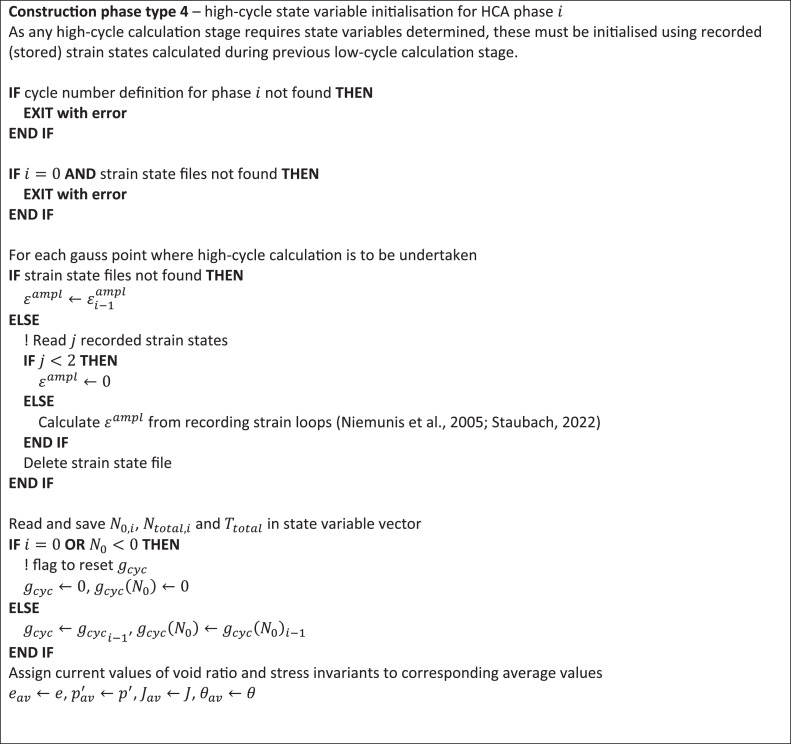
Integration flowchart for construction phase type 4 to initialise high-cycle state variables from calculation information stored during previous low-cycle calculation.

**Fig. 5:**
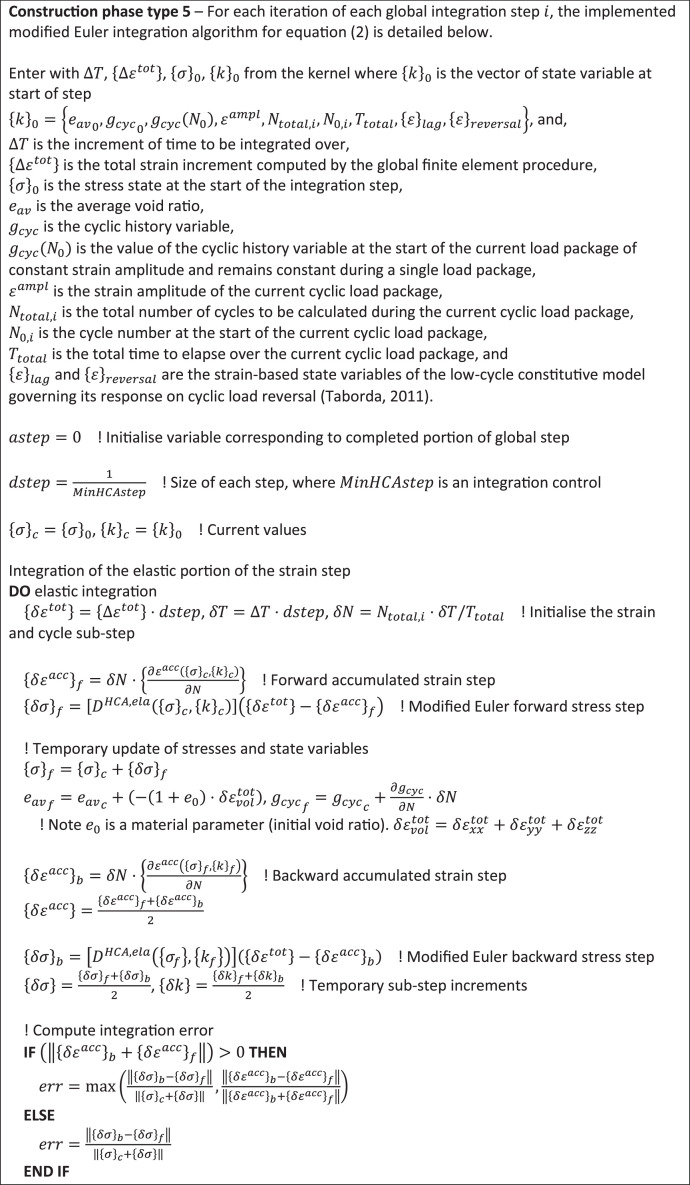

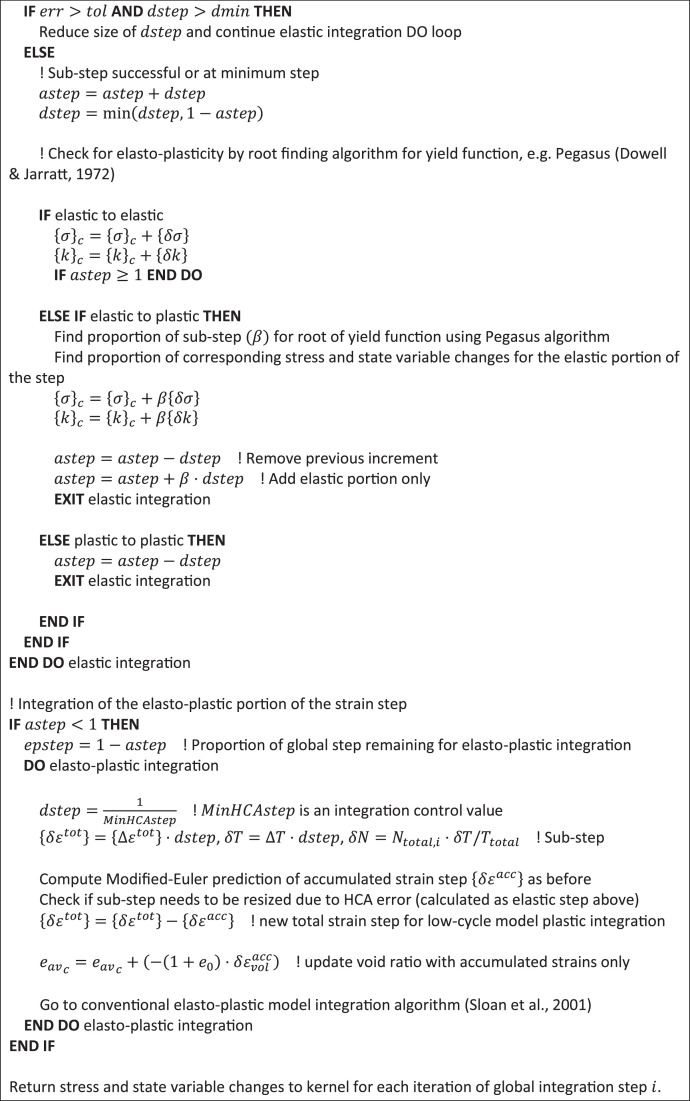
Integration flowchart for the two-stage modified Euler integration algorithm adopted to solve Eq. (2) during construction phase 5 (high-cycle analysis phases).

Fig. 4, Fig. 5 outline, in psuedo-code, operations carried out by the constitutive integrator (user-defined soil model) for construction phase types 4 and 5.

## Method validation

**Fig. 6:**
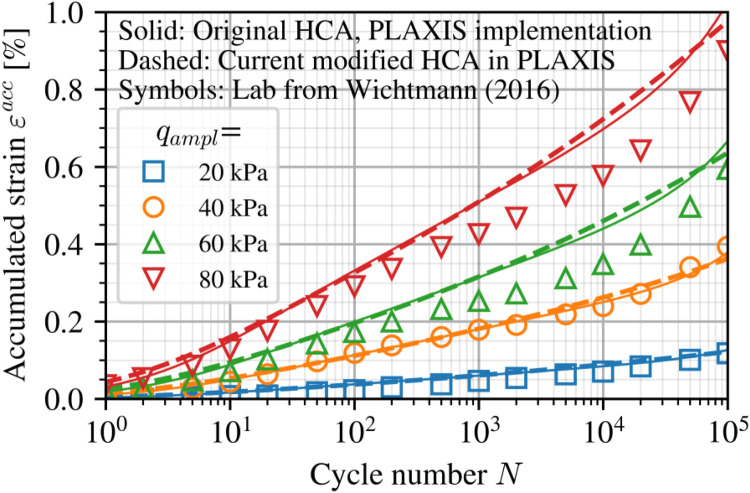
Comparison of current implementation of the original HCA formulation (solid lines), the modified functions (dashed lines) using parameters and laboratory test data (symbols) for Karlsruhe Fine Sand from the literature [8].

The current modifications and numerical implementation in PLAXIS were first tested against results from the original formulation [3] in its entirety without modifications and the laboratory tests on which it was based. This was developed based on a large set of tests on Karlsruhe fine sand carried out by Wichtmann [7]. Further details of those laboratory test results and the corresponding calibrated parameters (of the original model formulation) can be found in Wichtmann [[8]:p.134]. The same initial conditions of $p_{0}^{\prime }=200$ kPa, $q_{0}=150$ kPa and an average initial relative density of 62.5 %, corresponding to an initial void ratio $e_{0}=0.82$ are used herein. A comparison between laboratory test data and the simulated response for four different applied stress amplitudes $q_{ampl}$ is shown in Fig. 6. HCA parameters are taken from Table 4.2 in Wichtmann [[8]:p.135].

**Fig. 7:**
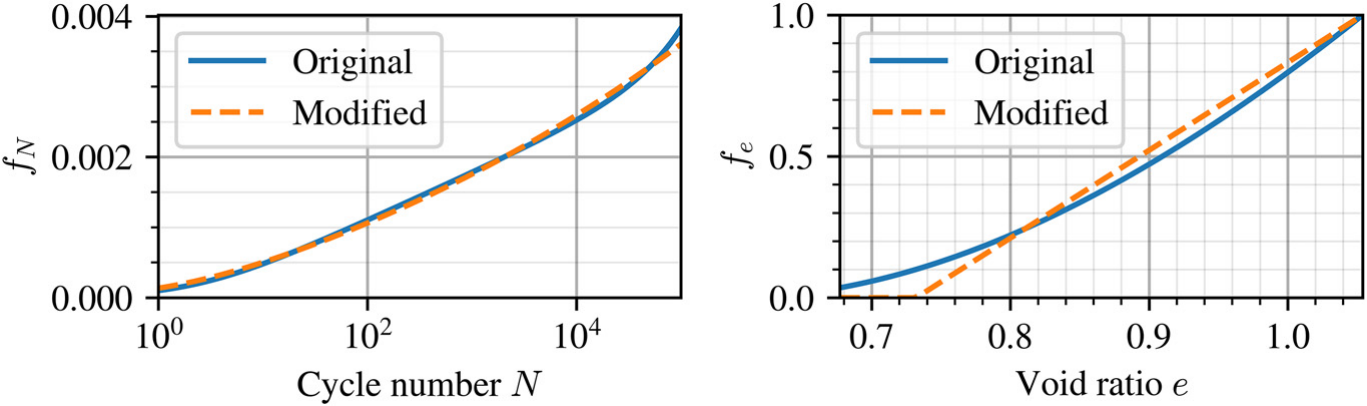
Modified functions compared to their original forms using re-calibrated $C_{N1},\;C_{N2},\;C_{e}$ parameters.

The main modifications made to the HCA functions, $f_{e}$ and $f_{N}$ (and correspondingly its derivative with respect to number of cycles ∂f/∂N), are then verified. Re-calibration of parameters $C_{N1},\;C_{N2}$ and $C_{e}$ is carried out simply by fitting the modified forms against the original, as illustrated in Fig. 7. The same element test simulations were recomputed and shown as dashed lines in Fig. 6. The differences at the single element level are negligible and show the applicability of the modified functions to capture the same behaviour at the material point level.

**Fig. 8:**
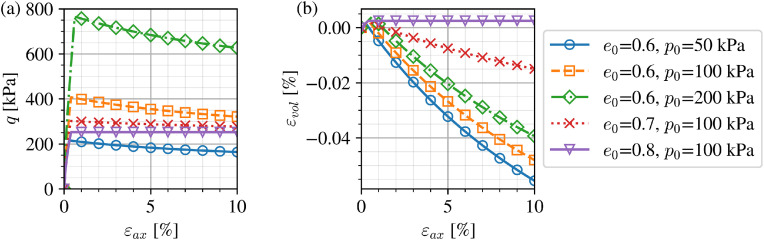
Drained isotropically-consolidated triaxial compression tests simulated using calibrated parameters for Dunkirk Sand in terms of (a) axial strain-deviatoric stress and (b) axial strain-volumetric strain.

**Fig. 9:**
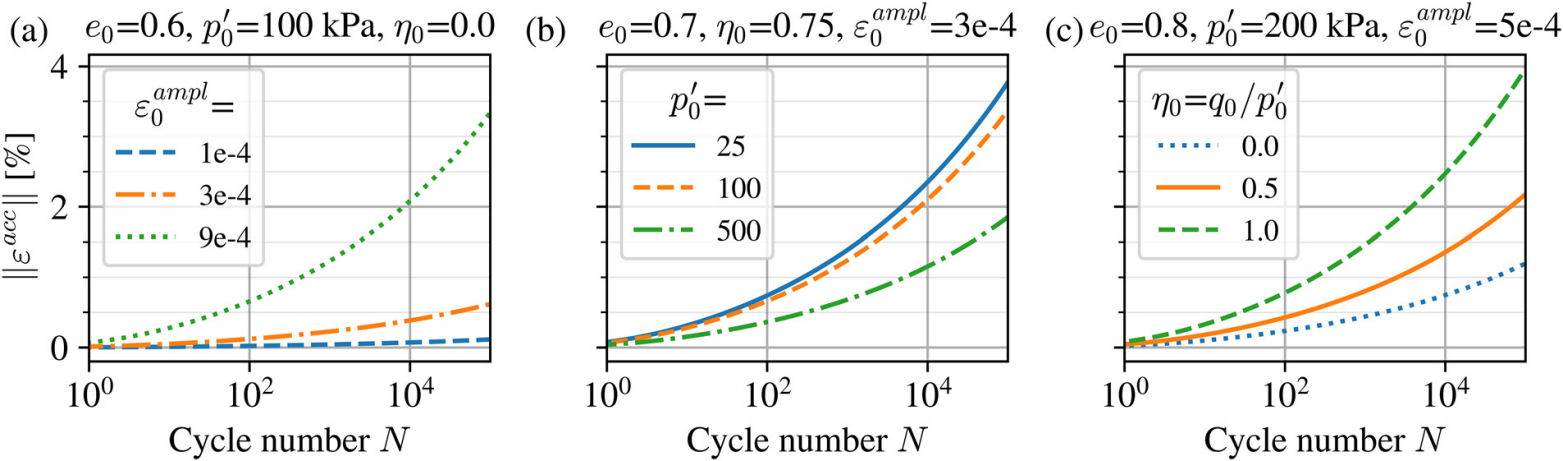
Cyclic drained triaxial test simulations using current calibrated parameters for Dunkirk Sand demonstrating the effect of (a) strain amplitude, (b) mean effective stress and (c) initial stress ratio.

To demonstrate the model implementation in PLAXIS for use in foundation design in a natural sand deposit, data from Dunkirk sand laboratory experiments [6] and in-situ site investigation [17] were examined and used to calibrate both low-cycle and high-cycle parts of the current model. As calibration results and discussion are outside of the scope of the current paper, a single representative set of parameters for the natural Dunkirk sand deposit (Table 2) was used in the model application. Representative monotonic and cyclic single element response at a wide range of stresses and densities are presented in Fig. 8, Fig. 9 respectively.

**Table 2:** Calibrated model parameters for Dunkirk sand.

Low-cycle parameter, see Taborda et al. [5]	$G_{\mathrm{ref}}$(MPa)	$m_{G}$	ν	a(%)	b	$R_{min}$	$e_{cs,ref}$	λ	ξ	$\phi {}_{cs}^{\prime }$$\left({}^{\circ }\right)$	k	l
Calibrated value	94.4	0.515	0.180	$2.21\cdot 10^{-2}$	0.911	0.104	0.910	0.135	0.179	33.9	2.34	3.91


High-cycle parameter			$C_{\mathrm{ampl}}$	$C_{N1}$	$C_{N2}$	$C_{e}$	$e_{\max}$	$C_{p}$	$C_{Y}$	$\phi_{\mathrm{mag}}^{\prime }$$\left({}^{\circ }\right)$	$\varepsilon_{\lim}$(%ε)	$\phi_{\mathrm{dir}}^{\prime }$$\left({}^{\circ }\right)$
Calibrated value			1.54	$1.95\cdot 10^{-4}$	0.17	0.13	0.91	0.15	1.64	33.9	0.1	26.8

The simulated monotonic drained response in Fig. 8 captures well the shear-volumetric coupling trends typical across a large range of densities and confining stresses. Denser and less confined sands (corresponding to more negative values of the state parameter ψ) exhibit greater dilation on shearing and greater softening in stress-strain space with both characteristics well reproduced by the model. The calibrated model's cyclic strain accumulation trends for a similarly wide range of density and stress conditions matches the same trends seen in drained cyclic triaxial laboratory tests [6,8,18] in Fig. 9.

**Fig. 10:**
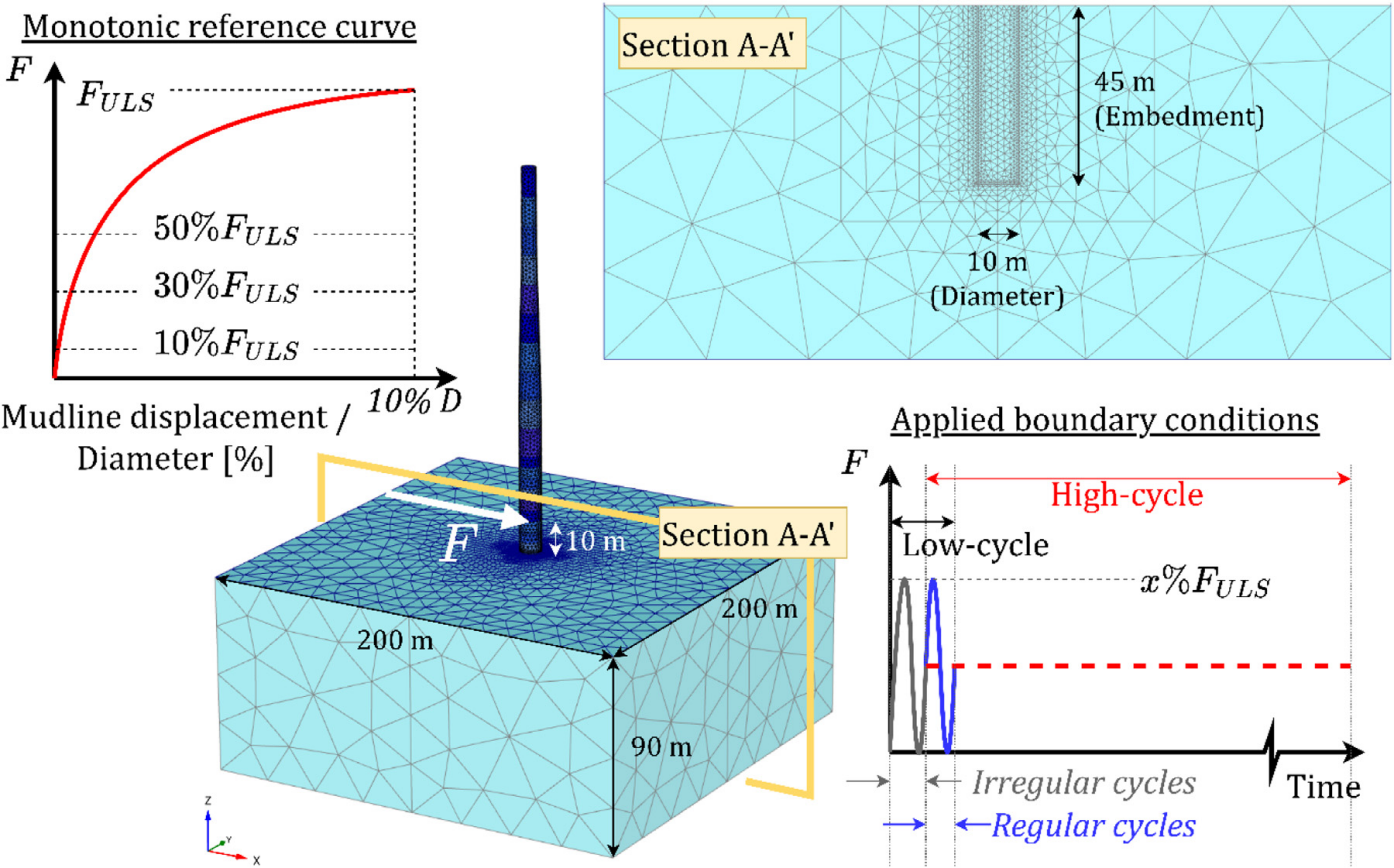
Schematic of the boundary value problem, boundary conditions and mesh employed.

Finally, the current implementation is verified for use in a boundary value problem in PLAXIS using a reference monopile foundation, with a diameter of 10m and an embedment of 45m, supporting a 15MW offshore wind turbine, the details of which can be found in Gaertner et al. [19]. Three analyses at different load levels are performed, where 100,000 cycles are applied at 10 metres above mudline level to simulate long-term wave loading. The structure is modelled using 6-noded quadratic shell elements with an isotropic linear elastic material with E=200 MPa and ν=0.26 following specifications in Gaertner et al. [19]. The soil-structure interface is explicitly modelled using 12-noded zero-thickness quadratic interface elements with a Mohr-Coulomb failure criterion defined by the angle of shearing resistance at critical state of the adjacent soil. The 200 m by 200 m by 90 m deep soil domain was assumed fully drained and discretized using 52,590 10-noded tetrahedral elements, resulting in 210,360 integration points. The mesh is highly refined in areas likely to experience concentrated changes in stress, such as those close to the pile. A hydrostatic initial pore pressure profile, a uniform relative density of 60 % and $K_{0}=0.4$ are assumed for the current offshore design scenario. Fig. 10 illustrates the employed mesh and applied loading boundary conditions.

**Fig. 11:**
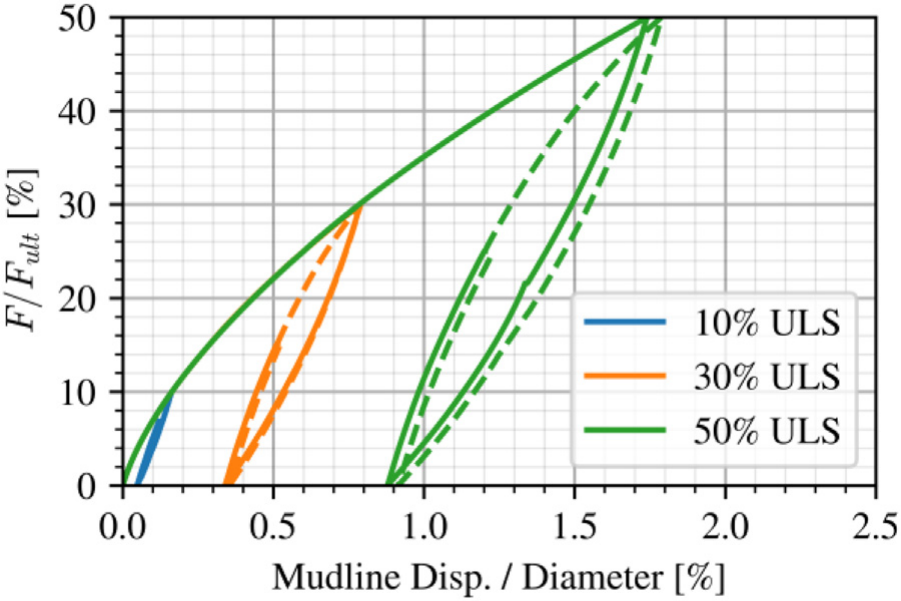
The low-cycle load-displacement response at three load levels plotted at mudline level (solid: irregular cycles; dashed: regular recorded cycles).

The computed low-cycle response at mudline level is shown in Fig. 11. Mudline displacement is normalised by the pile diameter (10 m) and the applied load F by the reference load at 10 % diameter displacement at the same level, as defined in Fig. 10. Hysteretic damping at the global level is captured during these cycle-by-cycle analysis phases.

**Fig. 12:**
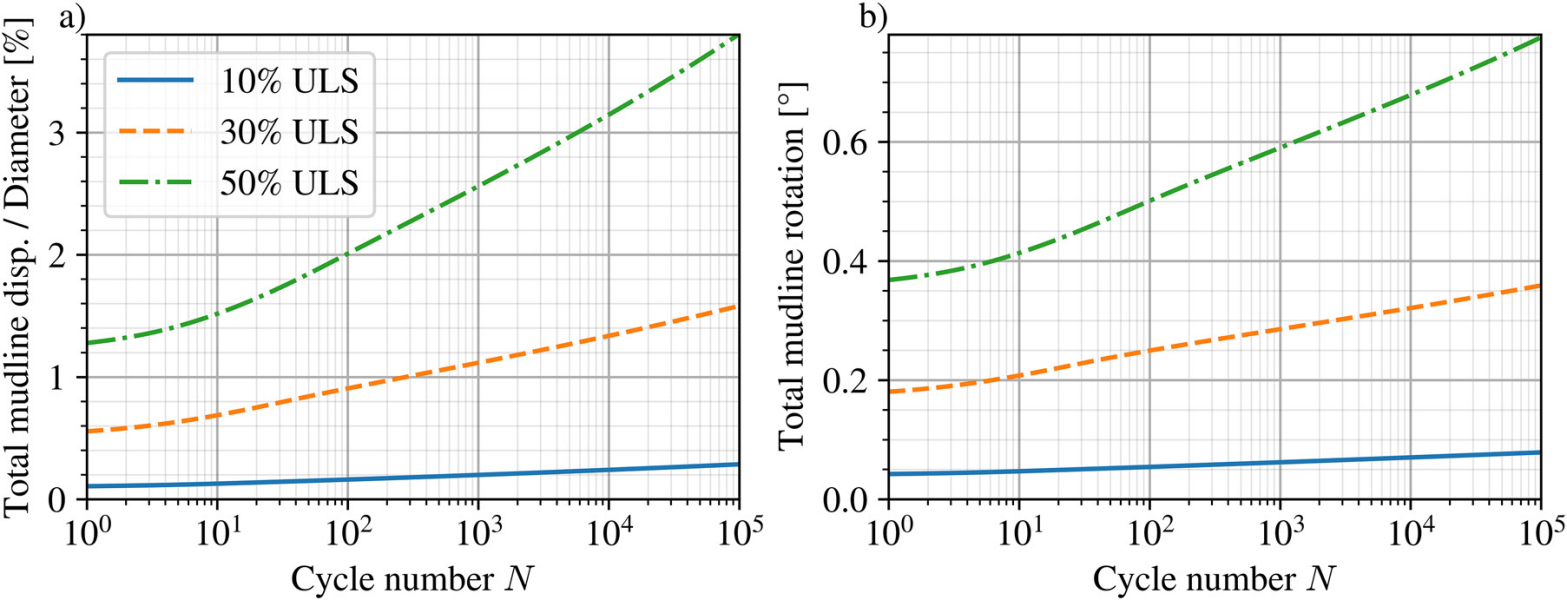
a) normalised displacement and b) tilt at pile head for the reference monopile with number of cycles.

The subsequent high-cycle analysis phase was computed up to 100,000 cycles and the pile head response is shown in Fig. 12 for normalised displacement and rotation at mudline. Total displacement and its rate of increase with number of cycles is observed to grow non-linearly with applied load level.

**Fig. 13:**
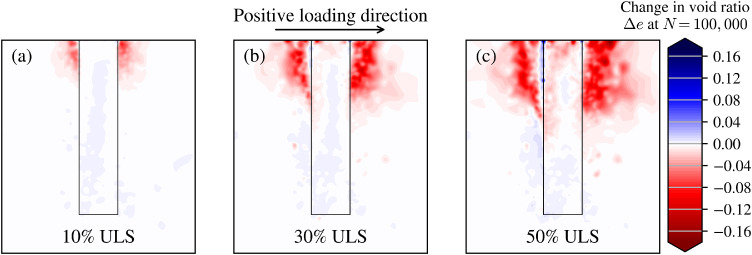
Contours of void ratio changes due to long-term cycling around the monopile foundation at three load levels. Negative void ratio change denotes compaction.

Fully modelling the soil continuum as finite elements allows an assessment of the underlying mechanisms driving the global foundation response. Changes in void ratio due to 100,000 applied loading cycles at the three load levels is shown in Fig. 13 for section A-A’ (indicated in Fig. 10). Increased densification in the predominant loading direction is seen with increasing load level.

## Limitations

The current framework and its use in finite element analyses requires inputs of regular load cycles about some mean cyclic load and cyclic amplitude. Direct computation of an arbitrary load varying with time is not possible without idealisation or transformation of the time series into discrete cyclic load packages. Load idealisation techniques such as rainflow counting have traditionally been suggested and the applicability of ‘Miner's rule’ has historically been assumed for drained sands [20]. However, recent studies [21,22] suggest that further investigation is required to fully quantify the effect of varying load amplitudes on strain accumulation. Additionally, the effect of loading rate is assumed negligible for sands subject to the types of environmental loading considered.

The current implementation extends the original method with a practice oriented constitutive model where each calibration step is site-specific and physically meaningful. The resultant model captures well the material's average strain accumulation and stress relaxation at a range of densities, void ratios, mean effective stresses and deviatoric stress ratios with number of cycles at the elemental level using a single set of parameters. Site-specific overconsolidation can be accounted for in the calibration by aligning laboratory testing using samples prepared to the assumed or assessed in-situ overconsolidation ratio.

In the global geotechnical problem, material states are not explicitly controlled but a product of the finite element calculation. Using the proposed approach and modifications, no recalibration is required due to the evolving material state during long-term cycling. In contrary, equivalent or degraded stiffness type approaches to the same problem [23,24] are highly dependent on the element tests selected for calibration.

## Ethics statements

This work does not involve human nor animal subjects. No data was collected from social media platforms.

## CRediT author statement

**Pishun Tantivangphaisal**: Conceptualisation, Methodology, Software, Validation, Writing – Original draft **David M G Taborda**: Software, Writing – Review & Editing, Supervision **Stavroula Kontoe**: Writing – Review & Editing, Supervision.

## Declaration of competing interest

The authors declare that they have no known competing financial interests or personal relationships that could have appeared to influence the work reported in this paper.

## Data Availability

Data will be made available on request.
